# Dynamic Visuomotor Transformation Involved with Remote Flying of a Plane Utilizes the ‘Mirror Neuron’ System

**DOI:** 10.1371/journal.pone.0033873

**Published:** 2012-04-20

**Authors:** Daniel E. Callan, Mario Gamez, Daniel B. Cassel, Cengiz Terzibas, Akiko Callan, Mitsuo Kawato, Masa-aki Sato

**Affiliations:** 1 ATR Neural Information Analysis Laboratories, Kyoto, Japan; 2 NICT Multimodal Communication Group, Kyoto, Japan; 3 ATR Computational Neuroscience Laboratories, Kyoto, Japan; Federal University of Rio de Janeiro, Brazil

## Abstract

Brain regions involved with processing dynamic visuomotor representational transformation are investigated using fMRI. The perceptual-motor task involved flying (or observing) a plane through a simulated Red Bull Air Race course in first person and third person chase perspective. The third person perspective is akin to remote operation of a vehicle. The ability for humans to remotely operate vehicles likely has its roots in neural processes related to imitation in which visuomotor transformation is necessary to interpret the action goals in an egocentric manner suitable for execution. In this experiment for 3^rd^ person perspective the visuomotor transformation is dynamically changing in accordance to the orientation of the plane. It was predicted that 3^rd^ person remote flying, over 1^st^, would utilize brain regions composing the ‘Mirror Neuron’ system that is thought to be intimately involved with imitation for both execution and observation tasks. Consistent with this prediction differential brain activity was present for 3^rd^ person over 1^st^ person perspectives for both execution and observation tasks in left ventral premotor cortex, right dorsal premotor cortex, and inferior parietal lobule bilaterally (Mirror Neuron System) (Behaviorally: 1^st^>3^rd^). These regions additionally showed greater activity for flying (execution) over watching (observation) conditions. Even though visual and motor aspects of the tasks were controlled for, differential activity was also found in brain regions involved with tool use, motion perception, and body perspective including left cerebellum, temporo-occipital regions, lateral occipital cortex, medial temporal region, and extrastriate body area. This experiment successfully demonstrates that a complex perceptual motor real-world task can be utilized to investigate visuomotor processing. This approach (Aviation Cerebral Experimental Sciences ACES) focusing on direct application to lab and field is in contrast to standard methodology in which tasks and conditions are reduced to their simplest forms that are remote from daily life experience.

## Introduction

This study explores the brain regions involved with dynamic visuomotor representational transformation. As a result of technological advances in the last several decades it is now common place for individuals to control vehicles and objects (real and simulated) remotely. Given this recent development it is unlikely that new neural processes have evolved to be able to accommodate this ability. Rather it is more plausible that neural processes already in place and utilized for other functions such as manipulation of tools are used for remote vehicle operation. The primary adaptive mode of perceptual-motor control is carried out in an egocentric body centered reference frame (1^st^ person perspective). The ability to utilize a remote reference frame (3^rd^ person perspective) for perceptual-motor control requires an additional representational transformation into a body centered 1^st^ person perspective. This ability to make this representational transform is thought to embody imitation that allows a person to reproduce actions of another person seen from a different perspective to accomplish some goal [1]. The ability to remotely control objects likely has its roots in imitation learning where it is necessary to put oneself in the perspective of the individual conducting the task in order to be able to replicate the task successfully. This is especially true when the dimensions of the object (vehicle) are rotating about their axis in a different perspective of that of the observer. To maintain reference to the vehicle and the control dimensions some form of visuomotor transformation is necessary.

There have been a considerable number of brain imaging studies that have investigated visuomotor representational transformation in reference to imitation [2]–[5], perspective processing [6]–[9], mental rotation [10], [11] and visuomotor learning [12]–[16] tasks. Studies investigating imitation have identified the ventral premotor cortex and superior parietal cortex [1]–[5] as being active during both observation and execution of action (‘Mirror Neuron System’). Based on these findings it has been hypothesized that imitation involves the understanding of action goals based on processes that map the observed action onto the motor representation of the same action [4], [5]. Recent reviews using activation likelihood estimation (ALE) [1], [17] together suggest that premotor and parietal regions are involved with both observation and imitation of action constituting a ‘Mirror Neuron’ system. In order for imitation to be possible it is necessary to be able to carry out a visual perspective transformation between one’s own body coordinates (body schema – intrinsic coordinate system) and that of the individual being observed. Studies investigating perspective visuo-spatial transformations have implicated the temporo-occipital regions [6], [7], [8], precuneus, premotor cotrtex PMC bilaterally, cerebellum bilaterally, left temporal cortex, left inferior parietal lobule, and left occipitoparietal cortex [9]. A meta-analysis of studies of mental rotation indicate transformation-specific activity present in the precuneus (temporo-occipital region), the PMC, Broca’s area (BA 44, 45,46), anterior cingulate, and fusiform gyrus [10]. Mental rotation of body parts (body schema) [11] has been shown to activate left PMC, left superior parietal cortex SPC, left inferior pariegal cortex IPC, and cerebellum. Many of the same regions involved with imitation, perspective processing and mental rotation have also been found in visuomotor learning experiments. For example, various studies investigating visuomotor learning of joystick movement transformation (rotation of visual feedback by a certain number of degrees) have implicated the cerebellum [13], [16], the posterior parietal cortex [15], [16], and the premotor cortex [12], [18]. It is maintained that activation in these areas, especially the cerebellum, is involved with predictive control utilizing internal models to learn the use of a new tool [13], [18]. (Also see [19] for a review on the computational mechanisms of sensorimotor control).

Previous studies investigating representational transformation related to perspective differences and mental rotation have several limitations: Most previous studies utilize tasks that are very simple and far remove from real-world experience. The response of the subject is usually via a button press to represent some complex motor task rather than directly engaging in the complex task; Subjects are often required to imagine the transformations rather than engaging in a task that requires them for success. The tasks are not engaging or dynamic. While visuomotor learning experiments (i.e. joystick movement transformation) utilize goal directed motor execution the tasks are usually to achieve a single trajectory target not complex perceptual-motor control. Furthermore, the transformation is not dynamically changing, as is the case in remote operation of an object (vehicle).

With the development of more powerful computers it is now possible to utilize realistic virtual environments for experiments exploring complex perceptual, motor, multimodal, and cognitive processes and still maintain flexible control over experimental variables. This approach is starting to be used more in neuroscience research [20]–[23]. This rich paradigm is in stark contrast to the standard experimental approach in which stimuli and tasks are reduced into very basic elements to more easily understand specific underlying neural processes. It is assumed that more complex perceptual, motor, and cognitive processes can be understood by combining these basic elements. While there are considerable advantages to the standard approach with regard to experimental manipulation and control there are also some major limitations. The tasks and stimuli used in standard experimental research are far removed from real-life experience. The only time we experience these conditions is in the laboratory. The assumption that processes investigated under these restrictive unnatural environments will scale up to normal real-world conditions may not be true or at a minimum would require investigating all the complex interactions between these processes. Another disadvantage of the standard experimental approach is that they are usually not engaging causing degradation of data due to fatigue. In standard experiments in which there are varying degrees of tedium in the various conditions one could imagine that differential brain activity mainly reflects the difference between being extremely bored in one condition versus being barely conscious in another condition. The new experimental paradigm using a rich virtual reality environment allows for investigation of complex engaging real-world tasks while maintaining experimental control over the task variables. This allows for more highly motivated subjects, less degradation of data due to fatigue, and can be more readily applied to brain-machine-interface applications under real-world conditions. One of the primary drawbacks of experiments using rich virtual reality environments is that there will be simultaneous activation of a considerable number of independent and interacting brain networks throughout the brain involved with processing various aspects of the complex task. To discern the neural processes underlying the task related variables under investigation it is important to have conditions that control for extraneous task and stimulus variables that serve as confounds. With the ability to manipulate aspects of the virtual environment engaging experiments can be designed that have experimental control to investigate specific neural processes underlying complex perceptual, motor, and cognitive functions.

**Figure 1:**
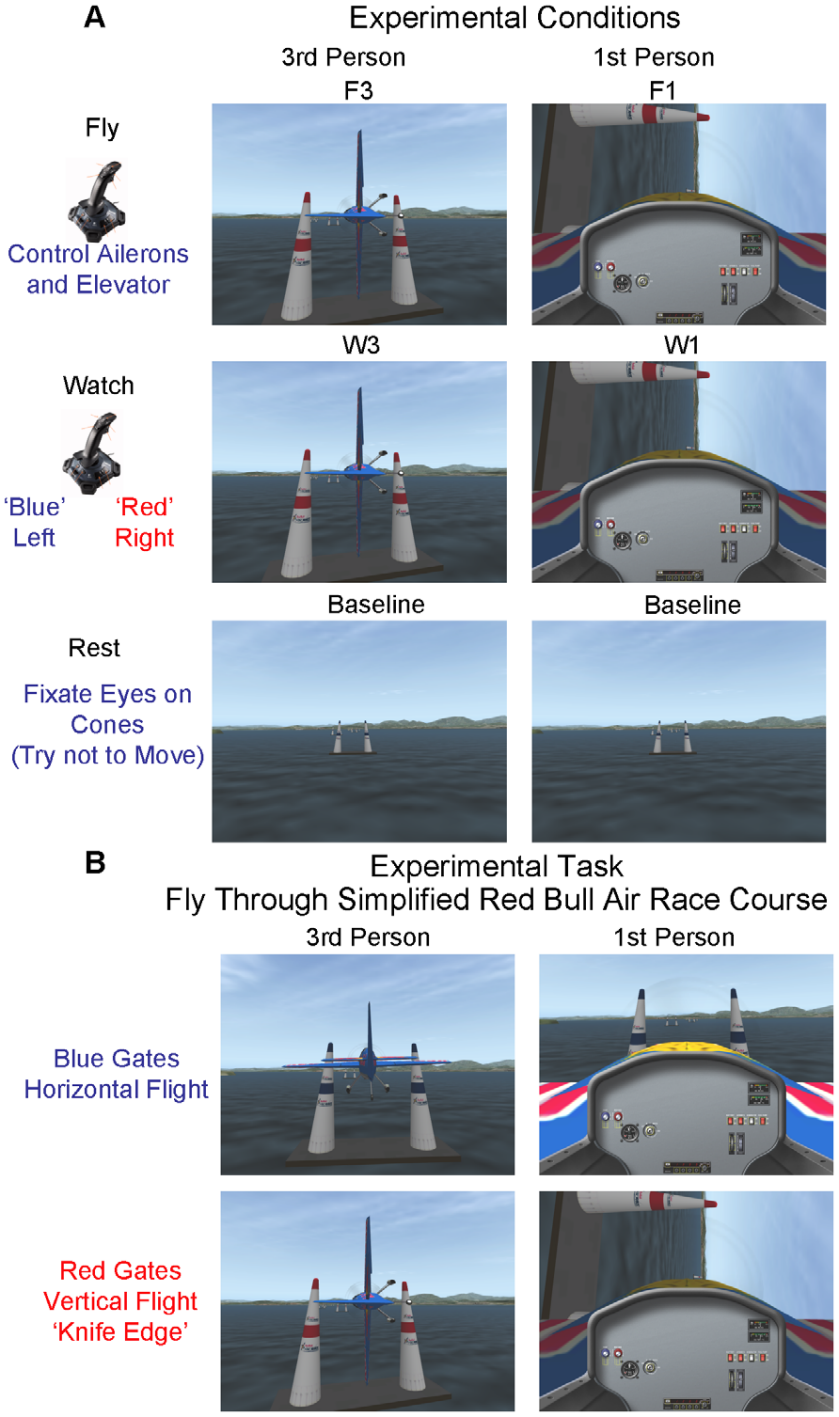
Experimental conditions and tasks. A: Experimental conditions consisted of flying the plane in 3^rd^ person (F3) and 1^st^ person (F1) perspectives as well as watching the flight of the plane while carrying out a simple motor task (move control stick to the right or left depending on color of the cones the plane is passing through) in 3^rd^ person (W3) and 1^st^ person (W1) perspectives. There was also a baseline condition where the subjects fixated their eyes on a pair of static cones and were instructed not to move. B: The task consisted of flying a plane through a simplified Red Bull Air Race course. Subjects were required to fly through red cones in a vertical manner in between the indicated marks with respect to altitude. Subjects were required to fly through blue cones in a level horizontal manner between the indicated marks with respect to altitude.

In this study we investigate brain regions involved with visuomotor representational transformation for remote operation of a simulated airplane on a complex perceptual motor task. It is hypothesized that remote operation (3^rd^ person perspective) utilizes additional neural processes to map between ones own body coordinates (body schema – intrinsic coordinate system) and that of the object under remote control. The experiment utilizes 4 experimental conditions and a baseline rest condition. The experimental task is to fly an airplane through a series of paired cones (resembling that of a simplified Red Bull Air Race course) in a specified altitude and orientation in 3^rd^ person chase perspective (F3) and 1^st^ person (inside the airplane cockpit) perspective (F1) (Figure 1A). Additional conditions are included in which subjects watch simulated flight in 3^rd^ person chase perspective (W3) and 1^st^ person perspective (W1) while carrying out a simple non-piloting perceptual motor task (Figure 1A). This experiment utilizes an engaging real-world task to investigate neural processes related to representational transformation while carrying out complex perceptual motor active control as well as during observation of the same action. Based on studies investigating imitation, perspective processing, mental rotation, and visuomotor learning (reported above) it is hypothesized that brain regions related to representational transformation for active remote control (3^rd^ person perspective) will include parietal areas, the premotor cortex, the cerebellum, and tempo-occipital visual areas. The foundation for remote operation is thought to be related to imitation learning. As such, activity in the ‘Mirror Neuron’ system including the premotor and parietal cortex is predicted to be greater for conditions requiring representational transformation (3^rd^ person greater than 1^st^ person) for both the action execution (flying) and the action observation (watching) tasks (however, at greater levels for the action task). If the ‘Mirror Neuron’ system is not involved in representational transformation and only mediates processing of action goals one may predict no difference in these regions for 3^rd^ person versus 1^st^ person conditions. Or even the opposite prediction of 1^st^ relative to 3^rd^ person showing greater activity in motor related regions because the motor representation of the action is more readily available [8].

Even though the visual information is controlled for in the respective (1^st^ and 3^rd^ person) flying and watching conditions the visual information between the 1^st^ and 3^rd^ person views does have considerable differences. This is evident in Figures 1A–B especially when approaching the red cones (as well as Movie S1 and S2). In the 1^st^ person view, the cockpit of the plane remains static while the environment changes its relationship in correspondence to the attitude of the plane with reference to the Earth. In the 3^rd^ person view the horizontal axis of the environment remains static while the plane moves. It should be noted that the forward motion optic flow field is moving at the same rate for both the 1^st^ and 3^rd^ person perspectives. This is because the 3^rd^ person perspective is from a chase view directly behind the plane. The calculation of distance to the cones and altitude above the ground is likely to utilize the same mechanisms in 3^rd^ and 1^st^ perspective given the very similar optic flow patterns in the forward moving direction due to the chase perspective in the 3^rd^ person perspective. There are, however, considerable differences in the visual information with regards to rotation. In the 1^st^ person perspective the optic flow field has rotation during maneuvering the plane, whereas, in the 3^rd^ person perspective the optic flow field remains in a fixed orientation and the plane itself rotates. Additionally the view in the 3^rd^ person chase perspective shows a larger part of the ‘world’ and is from a point behind the plane allowing the cones to be more easily viewed. The view of the ‘world’ in the 1^st^ person perspective is from inside the plane and is partially occluded by the cockpit. These differences in visual information presented in 3^rd^ and 1^st^ person perspective are likely to result in differential brain activity in visual processing regions. One may predict greater activity for the 3^rd^ person perspective in ventral visual areas involved with object perception (such as lateral occipital cortex LOC regions [24]–[26]) as the plane is viewed from behind. On the other hand one may predict greater activity in brain regions involved with processing motion, such as medial temporal MT region [27] for the 1^st^ person perspective because of the stronger motion stimulus present when tilting the view of the world.

While it logically follows that executing actions in a complex perceptual motor task will activate brain regions that are not present during observation of the action it may also be the case that observation of a complex perceptual motor task may differentially activate some brain regions to a greater extent. In this study we explored this possibility by determining activity that is greater for the 3^rd^ person watching condition over that of the 3^rd^ person flying condition as well as over the 1^st^ person watching condition. It is hypothesized that this contrast will reveal activity in brain regions involved with additional brain processes that are utilized for internal simulation during observation of action that are not used to the same extent when the action is being carried out and real visual-motor feedback is available. Candidate brain regions for these processes include the parietal cortex, frontal cortex, and cerebellum.

## Methods

### Ethics Statement

All subjects gave written informed consent for experimental procedures approved by the ATR Human Subject Review Committee in accordance with the principles expressed in the Declaration of Helsinki.

### Subjects

Thirteen 21- to 40-year-old (12 male, 1 female) right–handed subjects participated in this study. While sex differences have been shown to exist on some motor control tasks [28] (however, see [29] in which a null result of sex differences in brain activity during a visuomotor task has been reported) we do not expect the unbalanced number of male and female subjects to affect the overall results of our study. Although we cannot assess sex differences between the various conditions of our experiment, because only one female was included, it may be interesting to investigate this in the future.

### Procedure

**Figure 2:**
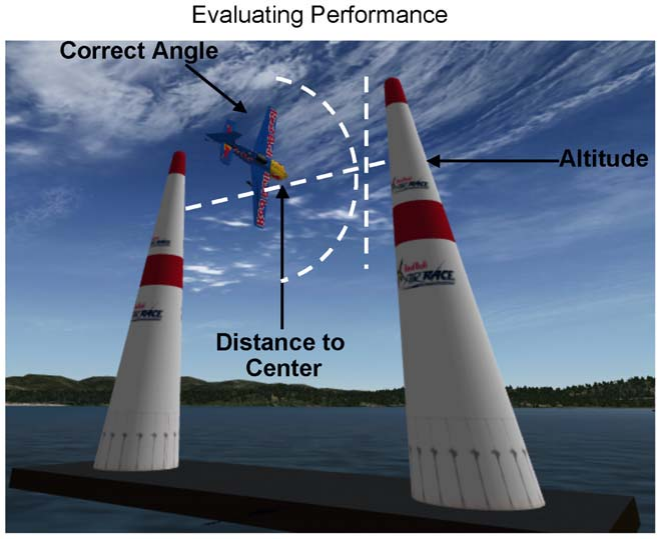
Evaluating task performance. Performance on the flying task was evaluated based on three measures: 1. Distance to the center of the cones when passing through the gates; 2. The distance to the target altitude denoted by the distance to the center between the colored bands on the cones; 3. Degrees to correct angle (0 degree target for level flight; 90 degree target for vertical flight).

The experimental task, using the X-Plane version 9.31 (Laminar Research) flight simulator, consisted of piloting an aerobatic airplane (Edge 540; Wing Span 7.43 meters; Average speed 285 km/hr; Developed for X-Plane by Rob Heap, Patrick Wheeler, and Paul Jones) through a simulated Red Bull Air Race course (Red Bull objects for X-Plane developed by Fred Ider) in either 1^st^ person (view as if sitting inside an airplane and directly controlling its pitch and roll) or remote 3^rd^ person chase perspective (view as if following behind the airplane while remotely controlling its pitch and roll). In both conditions the control stick is always oriented in reference to the plane. In the 1^st^ person flying condition (F1) body coordinates are the same as plane coordinates. In the remote 3^rd^ person flying condition (F3) body coordinates are only the same as plane coordinates in straight and level flight. For each of the flying conditions there were matched conditions in which the subject watched a movie of someone else flying the course in 1^st^ person (W1) or remote 3^rd^ person chase perspective (W3). The baseline condition consisted of a static picture of the Red Bull Air Race course (Figure 1A–B). The task in the flying conditions was to fly the plane through a series of large paired cones (separated by 14 meters) at a specified marked altitude range (16.5 meters target) and at a certain orientation (blue cones – horizontal ‘level’ flight; 0 degrees; red cones – vertical ‘knife edge’ flight; 90 degrees) (Figure 1B). The performance for each paired set of cones was determined by three measures (Figure 2): 1. The distance to the center of the gate; 2. The distance to the target altitude; 3. Degrees to correct angle (0 degree target for level flight; 90 degree target for vertical flight). If the altitude of the plane dropped below 5 meters above sea level it was designated as a crash and the plane was reset to the beginning of the course receiving a scoring penalty. In the watching condition the subjects moved the control stick to the left when going through a blue cone and right when going through a red cone (counterbalanced across subjects) (Figure 1A). Each of the conditions was pseudo randomly given in 25-second blocks with 5 seconds between blocks for instructions (The instructions were displayed on the screen for 2.5 seconds). The instructions consisted of Fly, Watch, or Rest. Within one block (trial) there was time to pass through 6 pairs of cones without crashing. The cones were arranged in the following order: 1 blue; 2 blue; 3 red; 4 red; 5 blue; 6 red. Each of the conditions was repeated 4 times within a single run lasting approximately 12 minutes (4 runs were presented). All subjects did receive approximately 1 hour of practice flying through the course a few days prior to the start of the experiment as well as about 10 minutes immediately prior to the start of the experiment. Previous experience with computer games was not recorded.

The behavioral performance for flying through the Red Bull Air Race course was assessed based on the mean value of three measures (See Figure 2). The three measures consist of 1. Distance to center between the two cones; 2. Distance to target altitude (denoted by the center between the colored stripes on the cones); 3. Distance to correct angle (Blue Cones = Level Flight 0 degrees target; Red Cones = Knife Edge Vertical Flight 90 degrees target). The scores for each measure were normalized such that 1 was the highest possible score denoting perfect performance and 0 was the lowest possible score. A maximum number of 6 Cones can be passed within the 25-second trial duration. The sum of each of the three measures for all cones passed was calculated (a crash counted as -3 points) and then divided by 6 (total number possible cones passed). Negative scores were counted as 0.

### fMRI Data Collection and Preprocessing

Visual presentation of the flight simulation was projected via mirrors to a screen behind the head coil that could be viewed by the subject by a mirror mounted on the head coil. An fMRI compatible control stick (NATA technologies) was used by the right hand of the subject to control the aileron deflection (roll left and right) and the elevator deflection (forward = pitch down and back = pitch up). The flight simulator was controlled via an UDP interface (100 Hz) to a computer running the experimental protocol in Matlab. Over 300 flight parameters of the airplane were collected at a rate of 100 Hz and included (latitude, longitude, altitude, roll, control stick X (aileron) and Y (elevator) deflection). Given the known latitude and longitude of the cones the performance through the course could be evaluated using these flight parameters.

The Siemens Trio 3T scanner was used for brain imaging at the ATR Brain Activity Imaging Center. Functional T2* weighted images were acquired using a gradient echo-planar imaging sequence (echo time 30 ms; repetition time 2500 ms; flip angle 80°). A total of 40 interleaved axial slices were acquired with a 4×4×4 mm voxel resolution covering the cortex and cerebellum. A single run consisted of 290 scans. The first 7 scans were discarded. T2 structural images, later used for normalization, were also collected using the same axial slices as the functional images with a 1×1×4 mm resolution. Images were preprocessed using SPM8 (Wellcome Department of Cognitive Neurology, UCL). Echo planar images EPI were unwarped and realigned. The T2 image was coregistered to the mean EPI image. The T2 images were acquired during the same fMRI session as the EPI images with the same slice thickness. Since the head was in approximately the same position it is thought that this will facilitate coregistration. The EPI images were then spatially normalized to MNI space (3×3×3 mm voxels) using a template T2 image and the coregistered T2 image as the source. Normalization was done using the T2 image rather than EPI because we believe it gives better results due to better spatial resolution. The images were smoothed using an 8×8×8 mm FWHM Gaussian kernel. Regional brain activity was assessed using a general linear model employing a boxcar function convolved with a hemodynamic response function (block design experiment). High pass filtering (cutoff period 128 seconds) was carried out to reduce the effects of extraneous variables (scanner drift, low frequency noise, etc). Auto-regression was used to correct for serial correlations. The 6 movement parameters were used as regressors of non-interest in the analysis to account for biases in head movement correlations present during the experimental conditions.

### fMRI Analysis

Contrasts between the various conditions were estimated for each subject using the general linear model. The parameter estimates of the analyses for each subject were used to conduct between subject random effects one-sample t tests for the contrasts of interest. The contrasts of interest include the following: Experimental conditions relative to rest (F3-rest; F1-rest; W3-rest; W1-rest); Flying relative to watching conditions (F3-W3; F1-W1); Watching relative to flying conditions (W3-F3); 3^rd^ person relative to 1^st^ person for flying and watching conditions (F3-F1; W3-W1). The contrast of F3-F1 (and W3-W1) assesses differences in perspective on the same task but does not control for differences in the properties of visual presentation. The contrast of F3-W3 (and W3-F3) assesses flying relative to watching differences for the same perspective but does not control for extraneous task differences such as difficulty, degree of motor control, degree of control stick movement etc… The simple motor task in the watching conditions (W1 and W3) is used to ensure that subjects are actually attentively observing the visual information presented. Without this condition subjects may close there eyes or not pay attention to the visual stimuli. It is not meant as a control for general task related variables and motor control processes. The general task properties and motor control necessary to fly the plane are essentially identical in the 1^st^ person and 3^rd^ person perspectives. Therefore the contrast of F3-F1 controls for general task related properties and motor control.

To assess the brain regions underlying processing of dynamic visuomotor representational transformation while performing a complex perceptual motor task that takes into account both general stimulus and task variables (including motor control) the intersection (conjunction) of significantly active voxels for the F3-F1 contrast and the F3-W3 contrast was determined. Similarly the intersection of active voxels for the W3-W1 contrast and the W3-F3 contrast was determined to assess brain regions underlying processing of dynamic visuomotor representational transformation while viewing a complex perceptual-motor task but performing a simple perceptual-motor task that takes into account both general stimulus and task variables. The intersection of the F3-F1 contrast and the W3-W1 contrast was conducted to determine brain regions that commonly are active for dynamic visuomotor representational transformation for both performing and watching a complex perceptual-motor task. To ensure that differences in the conjunction between F3-F1 and W3-W1 were not a result of differences in properties of visual presentation, the contrast of F3-W3 was further included in the conjunction (Both F3 and W3 contain the same type of visual stimulation). All random effects statistics in this experiment reflect a voxel level FDR threshold of p <0.05 to correct for multiple comparisons over the entire brain (SPM8). The anatomical coordinates reported in this study are given in Montreal Neurological Institute MNI units.

## Results

### Behavior

The results of a paired t-test revealed statistically significant better performance in the F1 (Mean = 0.42, SE = 0.036) over the F3 (Mean = 0.33, SE = 0.043) condition (T = 3.63, p<0.005, n = 13). The simple motor task of differentially moving the control stick to the left or right depending on the color of the gate being passed resulted in 100% performance for both the 1^st^ person and 3^rd^ person perspectives.

The overall deflection of the control stick was also evaluated to assess whether one condition utilized more movement. The control stick deflection was assed by taking the summed absolute difference in its position (x and y norm) approximately every 10 ms for each trial. The total control stick deflection did not significantly differ between the F1 (Mean 24.5, SE = 1.7) and the F3 (Mean = 23.6, SE = 1.5) conditions (t = 1.62, p > 0.1, n = 13).

### Brain Imaging

**Figure 3:**
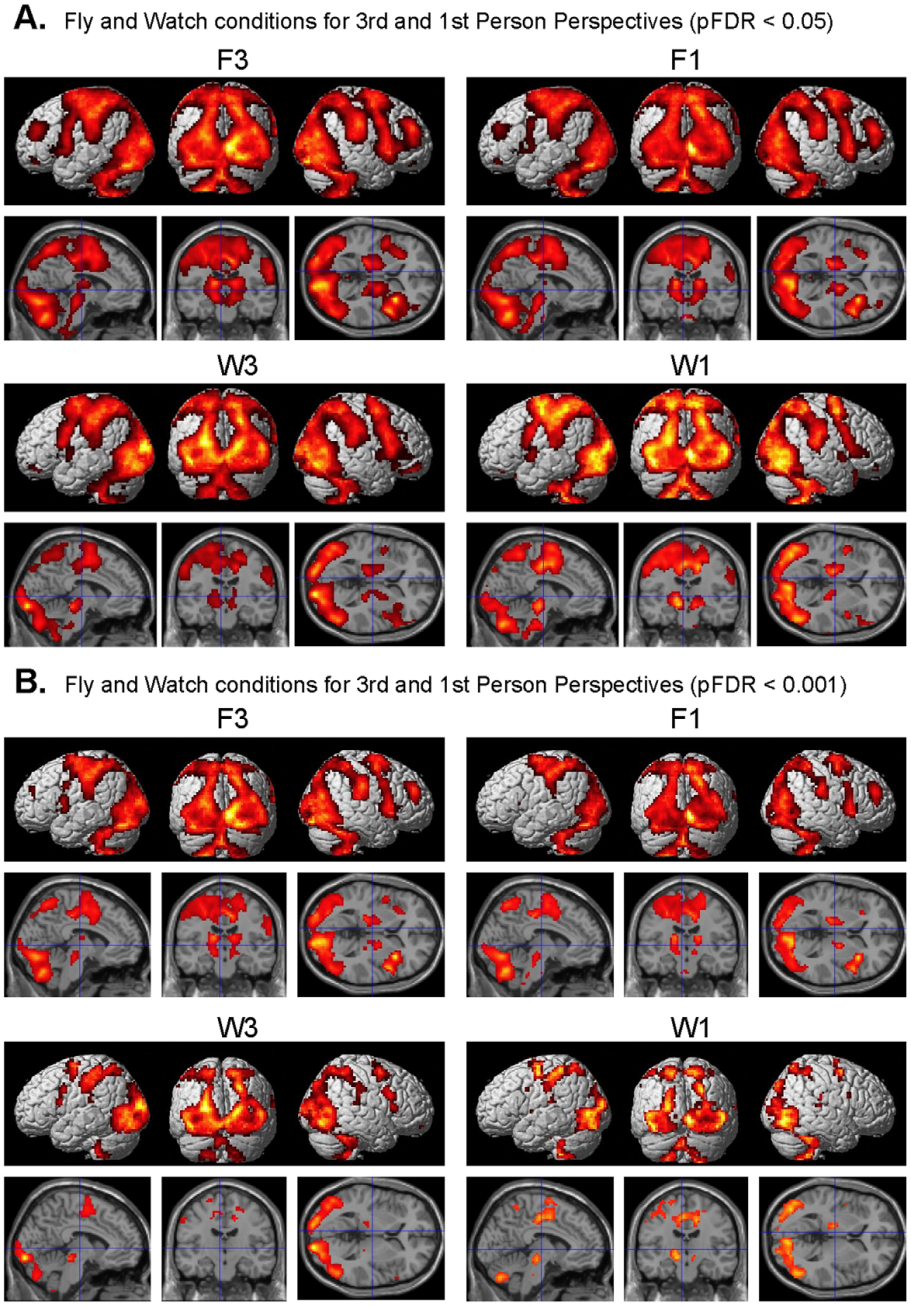
Differential activity rendered on the surface of the brain. A. Random-effects results rendered on the normalized brain of the 3^rd^ person flying condition (F3), the 1^st^ person flying condition (F1), the 3^rd^ person watching condition (W3), and the 1^st^ person watching condition (W1) relative to the baseline rest condition (pFDR <0.05 corrected for multiple comparisons). B. Same as above but using pFDR <0.001 corrected for multiple comparisons.

**Figure 4:**
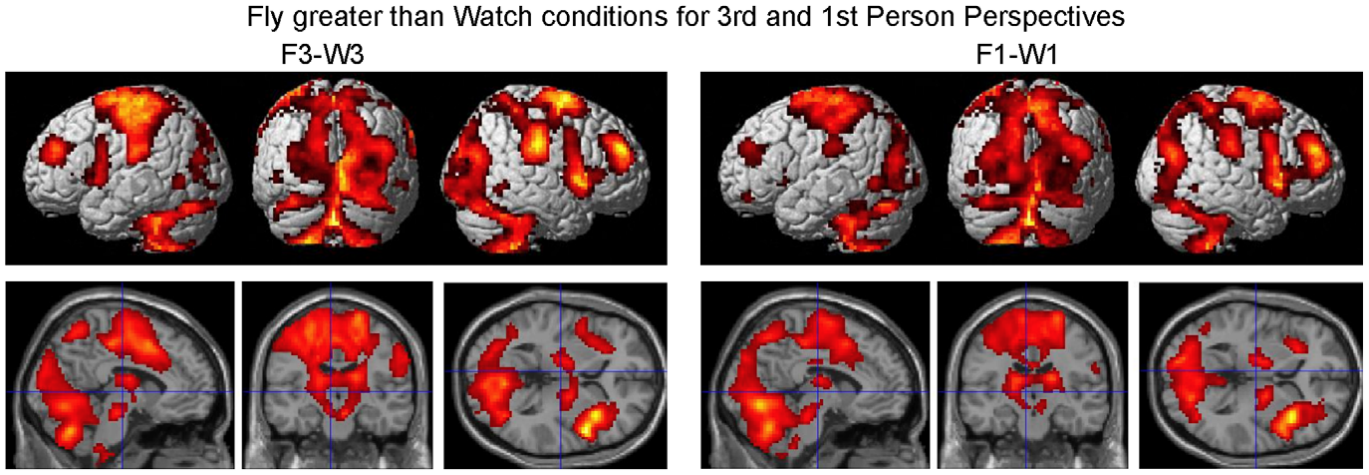
Differential brain activity between flying and watching conditions. Random-effects results of the contrast between flying and watching conditions for the 3^rd^ person perspective (F3-W3) and the 1^st^ person perspective (F1-W1) (pFDR <0.05 corrected for multiple comparisons).

The random effects SPM brain imaging results of the experimental conditions (F3, F1, W3, W1) relative to the rest condition are given in Figure 3A. All conditions show considerable overlap in the brain regions activated. These regions include wide spread activity across the occipital cortex, medial temporal MT, parietal cortex, somatosensory cortex, motor cortex, premotor cortex PMC, supplementary motor area SMA, middle frontal gyrus MFG, dorsolateral prefrontal cortex DLPFC, insula, cingulate, thalamus, basal ganglia, and cerebellum (pFDR <0.05 corrected for multiple comparisons). The presence of activity only in the left hemisphere motor strip (precentral gyrus BA4) is consistent with the use of the right hand for movement of the control stick. For the most part, the same brain regions remained to be active with a somewhat lesser extent using a threshold of pFDR <0.001 (Figure 3B). The exception being a great reduction or absence of activity in the left PMC for both the F1 and W1 conditions as well in the left MFG and DLPFC for the F1 condition. As can be seen in figure 4 the difference between the flying conditions and the respective watch conditions activated the same regions (albeit to a lesser extent) as the flying conditions relative to rest, with the exception of an absence of activity in the left PMv for the F1-W1 contrast (pFDR <0.05 corrected for multiple comparisons).

**Figure 5:**
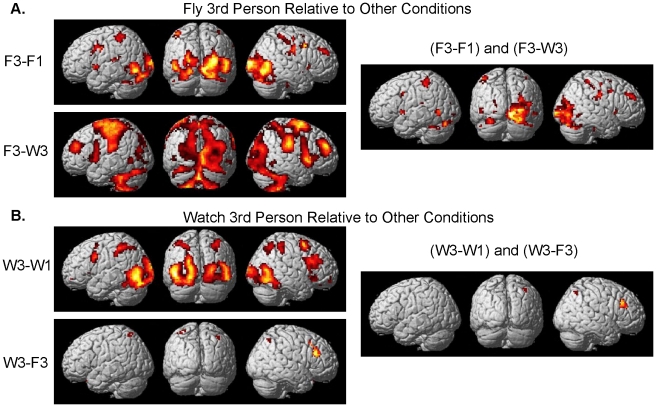
Brain activity specific to 3^rd^ person flying and watching conditions. A. Random-effects results rendered on the normalized brain of the 3^rd^ person flying condition relative to the other conditions as well as their conjunction (F3-F1) and (F3-W3) (pFDR <0.05 corrected for multiple comparisons). B. Random-effects results rendered on the normalized brain of the 3^rd^ person watching condition relative to the other conditions as well as their conjunction (W3-W1) and (W3-F3) (pFDR <0.05 corrected for multiple comparisons).

Differential brain activity of the contrasts focusing on F3 relative to the other conditions can be seen in Figure 5A and Table 1 (pFDR <0.05 corrected for multiple comparisons). It should be noted that because behavioral performance was greater for the F1 condition than for the F3 condition that the respective difference between the F3-F1 behavioral score for each subject was used as a nuisance variable in the random effects analysis of the F3-F1 contrast. The results of the contrast with and without using the nuisance variable are extremely similar. The brain regions showing differential brain activity for both the F3-F1 contrast and the F3-W3 contrast include bilateral activity in occipital cortex, MT, inferior parietal lobule IPL, ventral premotor cortex PMv, Broca’s, thalamus, basal ganglia, and cerebellum (Figure 5A and Table 1). Right hemisphere only differential activity for both F3-F1 and F3-W3 contrasts is present in the post central gyrus, pre central gyrus, dorsal premotor cortex PMd, and MFG (Figure 5A and Table 1).

**Table 1:** MNI Coordinates for Clusters of Brain Activity: Conjunction F3-F1 and F3-W3.

Brain Region	F3-F1 &F3-W3	Cluster Size	Peak T
Occipital BA17–18 R	24,–81,0	561	6.31
Occipital BA17–18 L	–21,–87,15	10	3.49
MT/MST R	45,–69,9	86	4.71
MT/MST L	–48,–66,3	60	5.36
LOC R	33,–78,–3	103	6.77
IPL BA40 R	45,–27,27	36	4.36
IPL BA40 L	–45,–39,57	66	4.3
PoG BA 2,3,5 R	36,–36,57	42	4.06
PoG BA 2,3,5 R	57,–15,33	40	4.03
PrG BA4 R	33,–15,57	18	4.01
PMd BA6 R	21,3,60	11	3.5
PMv BA6 R	27,6,36	41	5.36
PMv BA6 R	57,–15,33	76	4.03
PMv BA6 L	–54,6,39	10	4.92
SMA BA6 Pre-SMA L	–6,15,51	52	4.78
Broca’s BA44 45 R	45,15,3	18	3.81
Broca’s BA44 45 L	–57,6,3	11	3.93
Insula BA13 R	39,12,0	10	3.52
Insula BA13 L	–30,9,18	173	6.05
MFG BA8 R	24,36,33	56	6.58
MFG BA9 R	27,45,30	48	5.21
Thalamus L	–6,–12,6	75	5.13
Caudate R	21,–24,21	20	4.39
Caudate L	–21,–12,21	28	5.41
Globus Pallidus R	9,–3,0	20	5.68
Cereb Lob VIIa Crus 1 R	21,–81,–24	304	6.83
Cereb Lob VIIa Crus 1 L	–27,–78,–24	61	5.2

BA = Brodmann Area.

R = Right.

L = Left.

MT/MST = Medial Temporal/Medial Superior Temporal.

LOC = Lateral Occipital Cortex.

IPL = Inferior Parietal Lobule.

PoG = Post Central Gyrus.

PrG = Pre Central Gyrus.

PMd = Premotor Dorsal.

PMv = Premotor Ventral.

SMA = Supplementary Motor Area.

MFG = Middle Frontal Gyrus.

Cereb Lob = Cerebellar Lobule.

Figure 5B and Table 2 denotes the differential brain activity of the contrasts focusing on W3 relative to the other conditions (pFDR <0.05 corrected for multiple comparisons). Brain regions differentially activated more for the W3-W1 contrast include bilateral activity in the occipital cortex, MT, inferior temporal gyrus ITG, superior parietal lobule SPL, IPL, PMd, PMv, insula, orbital gyrus. Additional differential activity was present in the right post central gyrus, dorsolateral prefrontal cortex DLPFC, middle frontal gyrus MFG, and SMA. Greater differential activity for the contrast W3-F3 was present bilaterally in the SPL and IFG as well as in right PMd, DLPFC, and MFG. Brain regions showing activity for both the W3-W1 contrast and the W3-F3 contrast include the right SPL, DLPFC, and MFG (Figure 5B, Table 2).

**Table 2:** MNI Coordinates for Clusters of Brain Activity: Conjunction W3-W1 & W3-F3.

Brain Region	W3-W1 &W3-F3	Cluster Size	Peak T
SPL BA7 R	36,–63,51	6	3.77
DLPFC BA46 R	51,24,24	31	3.89
MFG BA9 R	54,30,36	34	6.1

BA = Brodmann Area.

R = Right.

SPL = Superior Parietal Lobule.

DLFC = Dorsolateral Prefrontal Cortex.

MFG = Middle Frontal Gyrus.

**Figure 6:**
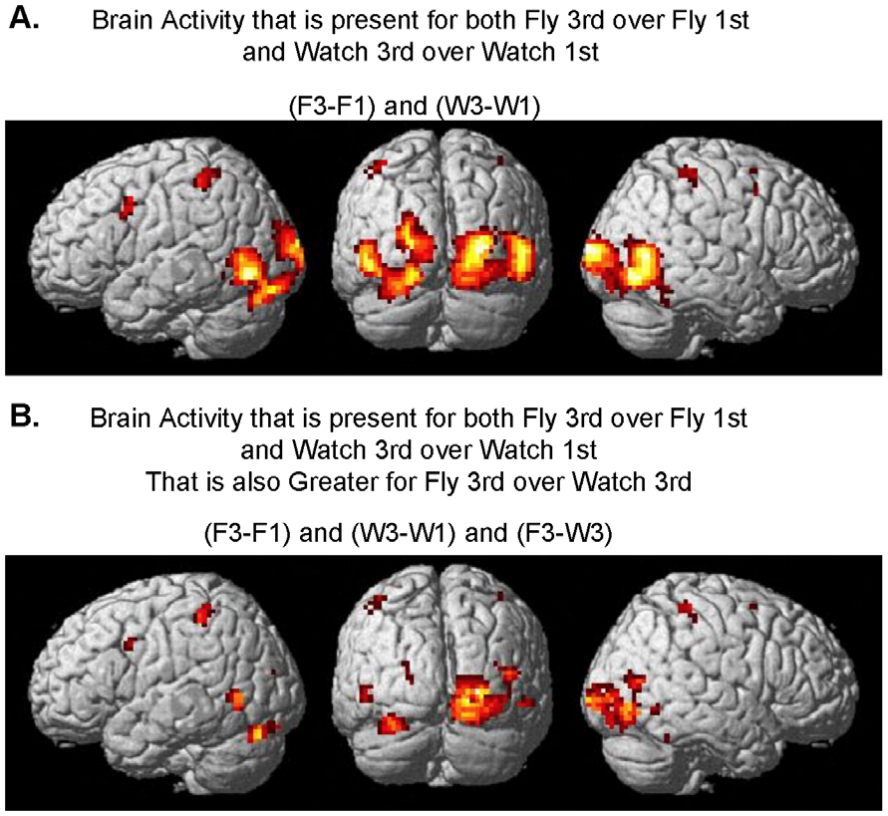
Differential 3^rd^ person brain activity present during both execution and observation of action. Random-effects results rendered on the normalized brain denoting ‘Mirror Neuron’ system characteristics in that the differential activity is present both for flying (execution) and watching (observation) conditions A. (F3-F1) and (W3-W1) with the additional constraint that the activity be greater for the flying (execution) condition B. (F3-F1) and (W3-W1) and (F3-W3) (pFDR <0.05 corrected for multiple comparisons).

Differential brain activity involved with processing 3^rd^ person perspective for both the flying and watching tasks is given in Figure 6A and Table 3 (pFDR <0.05 corrected for multiple comparisons). Brain regions showing significant differential activity for the random effects analysis of F3-F1 and W3-W1 include bilateral occipital cortex, MT, IPL, PMv, and cerebellum as well as right postcentral gyrus, and PMd (Figure 5A, Table 1). Figure 6B and Table 4 depict brain regions differentially active for the contrast of F3-W3 as well as F3-F1 and W3-W1. These regions include the occipital cortex, MT, IPL, right post central gyrus, right PMd, left PMv, and left cerebellum.

**Table 3:** MNI Coordinates for Clusters of Brain Activity: Conjunction of F3-F1 and W3-W1.

Brain Region	F3-F1&W3-W1	Cluster Size	Peak T
Occipital BA17–18 R	27,–102,12	452	7.55
Occipital BA17–18 L	–21,–90,–15	401	7.08
MT/MST R	54,–66,6	279	5.59
MT/MST L	–48,–66,3	234	5.36
LOC R	33,–78,–3	89	6.77
LOC L	–34,–78,–9	44	3.62
IPL BA40 R	39,–30,51	14	3.62
IPL BA40 L	–45,–39,57	38	4.3
PoG BA 2,3,5 R	36,–36,57	18	4.06
PMd BA6 R	21,3,60	4	3.5
PMv BA6 R	39,6,45	4	4.98
PMv BA6 L	–57,6,42	24	5.38
Cereb Lob VIIa Crus 1 R	12,–84,–15	52	6.21
Cereb Lob VIIa Crus 1 L	–27,–81,–21	40	5.51

BA = Brodmann Area.

R = Right.

L = Left.

MT/MST = Medial Temporal/Medial Superior Temporal.

LOC = Lateral Occipital Cortex.

IPL = Inferior Parietal Lobule.

PoG = Post Central Gyrus.

PMd = Premotor Dorsal.

PMv = Premotor Ventral.

Cereb Lob = Cerebellar Lobule.

**Table 4:** MNI Coordinates for Clusters of Brain Activity: Conjuntion of F3-F1 and W3-W1 and F3-W3.

Brain Region	F3-F1 & W3-W1 & F3-W3	Cluster Size	Peak T
Occipital BA17–18 R	21,–84,–3	234	5.91
Occipital BA17–18 L	–21,–87,15	9	3.49
MT/MST R	45,–69,9	47	4.71
MT/MST L	–48,–66,3	52	5.36
LOC R	36,–75,–6	62	5.5
IPL BA40 R	39,–30,48	18	3.45
IPL BA40 L	–45,–39,57	30	4.3
PoG BA 2,3,5 R	36,–36,57	9	4.06
PMd BA6 R	21,3,60	4	3.5
PMv BA6 L	–54,6,39	7	4.92
Cereb Lob VIIa Crus 1 R	42,–51,–27	4	3.72
Cereb Lob VIIa Crus 1 L	–27,–78,–24	56	5.2

BA = Brodmann Area.

R = Right.

L = Left.

MT/MST = Medial Temporal/Medial Superior Temporal.

LOC = Lateral Occipital Cortex.

IPL = Inferior Parietal Lobule.

PoG = Post Central Gyrus.

PMd = Premotor Dorsal.

PMv = Premotor Ventral.

Cereb. Lob. = Cerebellar Lobule.

## Discussion

The primary goal of this research was to determine brain regions underlying dynamic visuomotor representational transformations of the type necessary for remote operation of a vehicle or object. Unlike other experiments [12], [13], [14], [15], [16], [18] in which the visuomotor representational transformation to be learned is fixed (e.g. 90° rotation of visual feedback of joystick movement), in this experiment the degree of transformation is dynamically changing with the orientation of the plane (object). However, the motor control necessary to fly the plane is the same in both 1^st^ person and 3^rd^ person remote chase perspectives. This is to say that the plane flies exactly the same with the same control stick deflections regardless of the type of visual presentation. Consistent with the position that an egocentric body centered reference frame (1^st^ person perspective) is the primary adaptive mode of perceptual-motor control, behavioral performance on the Red Bull air race flying task (See Figure 2 for description of how performance was evaluated) was found to be better for the 1^st^ person perspective than the 3rd person remote chase perspective (F1 > F3).

The 1^st^ person and 3^rd^ person perspectives for both the flying and watching conditions showed considerable activity in visual brain regions, parietal cortex, PMC, SMA, MFG, DLPFC, pre and post central gyrus, insula, thalamus, basal ganglia, and the cerebellum (Figure 3A). The activity in parietal and premotor corticies was to be expected in the flying tasks but not as much in the watching task where only a simple motor task was employed (moving the control stick to the right or left based on the color of cones) that was not related to controlling the plane. Even with a more conservative threshold of pFDR <0.001 there is considerable activity in these same brain regions as was found with the more lenient threshold pFDR <0.05 with the exception of a reduction of activity in the left PMC for the F1 and W1 conditions (Compare Figure 3A and Figure 3B; also see below the discussion pertaining to greater activity in PMC for F3 over F1). This widespread activity shown in figures 3A–B likely reflects the multiple brain networks involved with carrying out as well as viewing the complex perceptual motor task of flying an airplane. The contrast of flying versus watching for 3^rd^ person and 1^st^ person perspectives (Figure 4) showed greater differential activity in these same brain regions suggesting that the activity is related to goal directed perceptual motor control even in visual areas rather than just a result of visual stimulation alone.

Brain regions thought to be involved with representational transformation for remote operation of a vehicle were determined by the voxels that were active for both the F3-F1 contrast and the F3-W3 contrast (Figure 5A, Table 1). The conjunction of these two contrasts helped to control for differences related to task (F3 and F1 employ the same flying task) as well as differences related to the visual aspects of the stimuli (F3 and W3 have the same visual perspective). Brain regions found to be involved with dynamic representational transformation consisted of visual areas, parietal cortex, pre- and post- central gyrus, PMC, SMA, insula, thalamus, basal ganglia, and cerebellum (Figure 5A, Table 1). These visual areas include the temporo-occipital region, LOC and MT region (overlapping with extrastriate body area EBA).

The conjunction (intersection of active voxels) of the contrast (F3-F1) and (F3-W3) controls for neural processes related to motor control of the flying task as well as for properties in visual stimulation. Because the same flying task is used in the 1^st^ and 3^rd^ person perspectives (F3-F1) and because the same type of visual stimulation is given for flying and watching conditions (F3-W3) it is maintained that the primary activation found in the conjunction analysis is related to processes involved with dynamic visuomotor transformation. While the right hemisphere activity in the conjunction contrast is fairly similar to the F3-F1 contrast it is quite apparent that the extent of left hemisphere activity is greatly reduced in the conjunction analysis compared to the F3-F1 contrast especially in visual regions including MT/MST (Figure 5A). The results suggest that much of the visual activity (F3-F1) in the left hemisphere is a property of visual stimulation as there is no significant activity found in this region for the contrast of (F3-W3) (Figure 5A, Table 1).

Greater activation in some regions of the visual cortex for 3^rd^ over 1^st^ person perspective may be explained by a larger field of view of dynamically changing visual stimulation because of the static view of the cockpit in the 1^st^ person perspective that occludes much of the optic flow visual information of the environment (Figures 5A–B; 6A). Consistent with our predictions, object-processing region LOC [24]–[26] in the right hemisphere showed greater activity in the 3^rd^ person than first person perspective most likely a result of the plane being present in the middle of the field of view (Figure 6A). However, the presence of activity in these visual regions when one controls for visual stimulation by contrasting the 3^rd^ person flying with the 3^rd^ person watching condition (Figure 6B, Table 4) suggests that these regions may be under attentional modulation [30] for processes related to aspects of dynamic visuomotor transformation.

A surprising result was the presence of greater activity in MT/MST for the 3^rd^ over the 1^st^ person perspective. It is well known the area MT and MST are involved with processing optic flow and visual motion [27]. Although the 3^rd^ and 1^st^ person perspectives both have an optic flow field that is moving at the same rate there are differences in the rotation of the field in the 1^st^ person perspective whereas, in the 3^rd^ person perspective the optic flow field does not rotate, but rather the plane in the center of the screen rotates within the moving optic flow field. One might predict greater activity in MT/MST in the 1^st^ person over 3^rd^ person perspective because it would seem that the view of the whole world tilting in the 1^st^ person perspective would produce much greater optic flow and motion stimulation than the plane viewed from behind in the 3^rd^ person perspective. However, in contrast to this prediction greater activity was found for 3^rd^ person over 1^st^ person perspective. This was true for both flying and watching conditions (Figures 5A–B, Tables 1–2). The activity in MT/MST also showed greater differential activity for 3^rd^ person flying over watching (Figure 5A and 6B, Tables 1 and 4), suggesting that it is not merely visual stimulus driven but may involve some form of attentional modulation and/or internal perceptual-motor processing involvement.

There are several potential explanations for why MT/MST activity may be greater in the 3^rd^ than 1^st^ person perspective. One possibility is that there are two motion fields in the 3^rd^ person perspective, the optic flow field of the chase perspective and the rotation of the plane. Consistent with this prediction it has been reported that there is greater activity in area MT with the number of distinct sources of motion in the visual scene [31]. The authors [31] suggest that MT may be involved with segmenting motion into independent sources, in this case the optic flow field and the rotating plane. Further evidence also implicates MT in multiple-object tracking [32] as well as local/differential flow due to object motion [31]. However, in contrast to the above predictions it has also been found that MT is more responsive to self-motion than object motion [33] which would suggest that there should be greater activity in the 1^st^ over 3^rd^ person perspective.

Another possible explanation for the greater activity for 3^rd^ person over 1^st^ person perspective is vestibular involvement and perhaps inhibition in MT/MST in the 1^st^ person perspective. One might expect the immersive environment of the 1^st^ person perspective to activate vestibular responses as well as motor reflexes to counter-act the perceived motion. The lack of true vestibular stimulation to multisensory brain regions involved with integrating vestibular and optic flow information may cause a reduction in response. Based on the principles of multisensory integration [34] a mismatch or lack of unimodal stimulation may cause a reduction of activity in multisensory processing regions. It is known that both visual and vestibular signals converge in MST [35]–[37]. Consistent with the above prediction based on multisensory integration it has been shown [38] that incongruous visual and vestibular stimulation results in a decrease in activation. However, the lack of finding MST neurons that have congruent rotation tuning for visual and vestibular stimuli [37] suggests that these neurons are not utilizing multisensory integration to enhance perception. It should be noted that since no experimental manipulations were made in this experiment concerning the vestibular system that this interpretation is highly speculative at this time and needs to be tested in future experiments.

In accordance with the hypotheses proposed in this study greater activity for 3^rd^ person over 1^st^ person perspective may reflect processes related to aspects of dynamic visuomotor transformation. In the 3^rd^ person perspective one needs to dynamically project the orientation of the control stick onto the same orientation that the plane is in currently. One needs to know this information to control the plane in three-dimensional space. Left and right deflection of the stick will roll the plane about its own axis. And forward and backward movement of the stick will rotate the plane in the pitch axis. When the plane is banked in a roll one utilizes the pitch control axis on the stick to maneuver the plane to the right or left with reference to the ground. Greater activity in the 3^rd^ person over the first person perspective in MT/MST may be a result of the known involvement of this area in object tracking [32] and heading perception [35] which is utilized to determine the orientation of the plane relative to the ground. This information is necessary for the neural processes involved with dynamic visuomotor transformation in the 3^rd^ person but not in the 1^st^ person perspective. It is interesting to point out that [39], [40] found activity (Figure 5A) in the EBA that was greater for 3^rd^ person allocentric perspective than for 1^st^ person egocentric perspective. The region they identified as EBA [39], [40] overlaps with that in our study identified as MT/MST. It is possible that activity in this region reflects processes related to transformation between ones own body space and that of the plane. Although [39], [40] explains activity in EBA as being responsible for processing other persons body parts it is also consistent to reinterpret their results as reflecting that perceiving an allocentric perspective requires a body related representational transform to process the information from an egocentric perspective. In relation to our study the results might suggest that EBA is not specific to just perception of body parts but more generally processing perspective changes with respect to ones own body. Future studies are needed to address whether the EBA is responding to body parts or to perspective changes (of body parts or objects operated by body parts) with respect to ones own body. Also related to the above hypothesis is the finding that the temporo-occipital region (near MT/MST) is involved with some sort of spatial transformation that is necessary during 3rd person more than 1^st^ person perspective [8]. This same region was also found by [6], [7] to be involved with egocentric body transformation consistent with the type of dynamic visuomotor transformation necessary to control the plane from a 3^rd^ person perspective. In future studies it would be interesting to examine brain activity that is parametrically modulated by the amount of dynamic visuomotor transformation related to the degree of rotation from body-centered coordinates.

It is maintained that the foundation for remote operation may be rooted in its use in imitation learning. It is hypothesized that the ‘Mirror Neuron’ system allows one to observe goal directed actions of others in relation to how one would execute the actions themselves [2], [4], [41], [42]. While the ‘Mirror Neuron’ system has often been discussed with reference to social context as a means to inform and support the mentalizing system (inferences about others’ abstract goals and beliefs) [3], [43], recent research suggests that the mentalizing system and the ‘Mirror Neuron’ system are not subservient of each other [44]. Rather, the ‘Mirror Neuron’ system is involved with imitation composing both observation and execution of actions [1]. If indeed the ability for 3^rd^ person remote operation of a vehicle is rooted in the same mechanisms as imitation learning one would predict greater activation in the ‘Mirror Neuron’ system for the 3^rd^ person perspective over that of 1^st^ person for both action execution (flying) and the action observation (watching) tasks (however, at greater levels for the action task). Consistent with this hypothesis was the finding of activation of brain regions involved with a motor execution system (parietal cortex, pre- and post- central gyrus, PMC, SMA, basal ganglia, and cerebellum) for flying in the 3^rd^ person chase perspective relative to the other conditions (Figure 5A, Table 1). Brain activity for the contrasts of both execution and observation for 3^rd^ relative to 1^st^ person perspective (F3-F1) and (W3-W1) (Figure 6A, Table 3) was present in ‘Mirror Neuron’ system areas including the right and left PMv, right PMd, left and right IPL and right post central gyrus. Activity was also present in the cerebellum and visual processing regions including MT. While the contrasts of (F3-F1) and (W3-W1) control for task differences they do not control for potential confounds resulting from the visual nature of the 3^rd^ and 1^st^ person perspective views. It was further predicted that execution would result in greater activity in ‘Mirror Neuron’ areas than observation. This contrast ((F3-F1) and (W3-W1) and (F3-W3)) (Figure 6B, Table 4) which does take into account possible differences in visual perspective views, revealed activity in left PMv, left and right IPL (extending into postcentral gyrus), right PMd, left and right Cerebellum, and visual processing regions including MT.

These results are consistent with the response properties characteristic of the ‘Mirror Neuron’ system. Previous studies have primarily implicated PMv and IPL as ‘Mirror Neuron’ system sites [1]–[5]. It should be noted that an activation likelihood estimation meta-analysis of fMRI studies [17] identified the PMd as well as the IPL and SPL as brain regions involved with imitation but not the inferior frontal gyrus IFG and the PMv. However, a more recent activation likelihood estimation meta-analysis over a larger number of studies did identify PMv and the IFG BA 44 (typically thought to reflect mirror neuron system) in the same left lateralized region we found in our study as being involved with both action observation and imitation [1]. The additional finding that observation of object compared to non object actions had stronger fronto-parietal activity [1] may be related to the plane being viewed as an object under control in the 3^rd^ person perspective to a greater extent than in the 1^st^ person where the experience was more direct control of moving ones own body. The IPL (more specifically the left IPL) has also been implicated in operations related to representational spatial transformations involving body-relative judgments [11]. Similarly, [2] also suggests left IPL involvement in body-part coding. Consistent with activity in the IPL and the results of [3] and [11] it may be conjectured that both observation and execution of flying in a 3^rd^ person chase perspective involves mental rotation (transformation) of the body to fit that of the airplane to be able to utilize the control stick in an egocentric reference frame. Studies [2], [4], [41], [42], [45] have also implicated the parietal cortex in processes related to imitation consistent with the involvement of this region as a part of the ‘Mirror Neuron’ system. A TMS study [46] further showed that parietal and PMC are involved with parallel processing of visuomotor information. The PMd has been implicated in tasks involving mental rotation [47]. Please see above for discussion of visual areas related to representational transformation.

The presence of bilateral cerebellar activity for both the 1^st^ person and 3^rd^ person perspectives relative to the baseline condition as well as to the watching only condition (Figures 4A–4B) may appear somewhat puzzling. One might expect right cerebellar activity if one is carrying out a sensorimotor task with their right hand [48]. However, the task in our experiment is very complex and requires spatial processing which is thought to be carried out in the left cerebellar hemisphere [48]. Greater activation in the right and left cerebellar hemispheres for the 3^rd^ person over 1^st^ person perspective (Figures 5A, 6A, 6B; Tables 1, 3, 4) may reflect processes involved with operation of the control stick in a dynamically changing visuomotor representational space. Because cerebellar activity is still present in the conjunction of (F3-F1) and (W3-W1) and (F3-W3) they can not be merely ascribed to differences in visual stimulation or differences in motor operation between the 3^rd^ and 1^st^ person perspectives. In support of the above hypothesis, studies have implicated the cerebellum in internal model processing of sensorimotor skills involving visuomotor transformation [13], [16] as well as in egocentric body related transformation [49]. It should be noted that bilateral cerebellar activity is also found in other experiments in which subjects learn a visuomotor transformation [50] utilizing only one hand to manipulate the joystick.

It should be pointed out that these findings are not likely a result of more movement in the 3^rd^ person condition because, as was stated above in the results section, there was an absence of a significant difference in control stick deflection between F3 and F1 conditions. Additionally the results are not just a reflection of differences in performance for the F3 and F1 conditions as this difference was modeled as a nuisance variable in the analysis (It should be noted that there was very little change in the overall results whether the nuisance variable was included or not).

While the results of this study (Figures 5A, 6A–B, Table 1, 3, 4) are consistent with the hypothesis that remote operation of a vehicle or object utilizes the ‘Mirror Neuron’ system that is involved with representational transformation of goal directed actions they are in direct contrast to those of [8]. In their study [8] it was found that the 1^st^ person perspective showed more activity in the left sensorimotor cortex than the 3^rd^ person perspective. The results were explained by suggesting that the 1^st^ person perspective has more direct access to motor representations [8]. As was pointed out by [8], the apparent inconsistency with their results with predictions of ‘Mirror Neuron’ theories may be due to the lack of clear goal-directed actions in the conditions of their experiment.

Also of interest in this study was the investigation of brain regions that may be used differentially for observation over execution of action in a 3^rd^ person perspective. The conjunction of the contrasts of W3-W1 and W3-F3 (Figure 5B, Table 2) revealed activity in right SPL and right middle frontal gyrus bordering on IFG BA45. The contrasts employed controlled for both task and visual perspective differences between the conditions. While the motor task of moving the control stick to the left or right based on the cones being passed required the subjects to observe the flight of the plane, the performance was perfect for both the 1^st^ person and 3^rd^ person conditions. This suggests that the involvement of right SPL and right MFG (Figure 5B, Table 2) was not due to differences in task difficulty between W3 and W1 but rather to differences in the observation of the plane in 3^rd^ person relative to 1^st^ person perspective. The finding of activity in the MFG bordering on IFG BA45 is consistent with findings that IFG BA45 is involved more with observation tasks than action tasks [1]. Together, the presence of activity in the right SPL and right MFG may reflect processes involved with internal simulation (prediction) of visuomotor feedback that is not necessary in the flying condition because it is directly available as a result of the relationship between actually moving the control stick and changes in the visual orientation of the plane on the screen.

There have been a number of studies utilizing virtual reality type stimulus presentation focusing on spatial navigation that may have relevance in interpreting our results [20]–[23]. While our task did not utilize spatial navigation in the same sense of traversing an environment based on spatial landmarks (such as in a maze) the task did require that the subject plan a trajectory up to and through the cones (banking either to the left or right for the red cones and horizontal flight for the blue cones) that would allow them to be in a position to be able to accurately traverse the next set of staggered cones while keeping in mind changes in aerodynamic stability (mainly lift) during steep banking (see Movies S1 and S2). Some studies focusing on spatial navigation have found activity in hippocampus [51] and entorhinal cortex [23] for spatial locations and the parahippocampus for views of landmarks [51]. We did not find activity in these regions in our study. This is not surprising given that spatial location and landmarks were not utilized for the navigation task demands for flying through the course. Rather the navigation task in our study was more in planning future trajectories. One aspect of our flying task that has been investigated is the use of spatial updating of objects during observer motion. Their study [20] identified the precuneus as being involved with the construction of updated representations and the PMd as being involved with context-dependent planning of motor actions. In our study precuneus activity was not present but PMd activity was present for both 3^rd^ and 1^st^ person perspectives for both flying and watching conditions (Figure 3A). Additionally, activity in the PMd for the conjunction of (F3-F1) and (W3-W1) and (F3-W3) (Figure 6B, Table 4) suggests the 3^rd^ over the 1^st^ person perspective may be utilized to a greater extent for context-dependent planning of motor control in flying through the course. Also of relevance to our results was the finding that spatial navigation through a virtual environment using a joystick activated left PMC and parietal areas (just as in our study; Figure 6B, Table 4) on a mental spatial distance calculation task than spatial navigation through the same environment using actual locomotion [22]. Consistent with the conclusions of our study the authors hypothesized that this activity reflects visuomotor processes related to tool manipulation. A considerable number of studies investigating tool use [18], [52], [53] have also implicated these same regions, PMC, parietal cortex, and the cerebellum, just as was found in our study (Figure 6B; Table 4).

Another potential explanation for the premotor and parietal activity in our study may reflect neural processes related to the use of a joystick as a tool to manipulate an object that does not require dynamic visuomotor transformation. While it may be possible to do this when there is no rotation about the axis of the control dimension (such as in video games like Pacman, Space Invaders, etc…) this was not the case in this experiment in which the object under control was rotated on the visual display but the physical joystick remained in relation to the subject. In many applications such as robotic surgery and operation of unmanned aerial vehicles the visual display is primarily given from a 1^st^ person perspective for ease of operation (as well as because of technical limitations). With relevance to teleoperation of robotic arms by joysticks guided by remote imaging sensors (3^rd^ person perspective) that are misaligned with reference to the operator, NASA scientists have determined that a kinesthetic cue of aligning the non-used hand in the orientation of the misalignment rotation reduces control disturbances by up to 64% [54]. This result is consistent with the hypothesis that operation of a vehicle from the 3^rd^ person perspective utilizes a visuomotor transformation into a self-based egocentric perspective to facilitate performance.

While the conjunction analyses in this experiment were utilized to control for neural processes related to motor control of the flying task as well as for properties in visual stimulation it is still possible that the activity found in our experiment reflects processes other than those related to dynamic visuomotor transformation. It is likely that the attentional load in flying is substantially greater than in the watching condition. It is however less clear what attentional differences may exist between 3^rd^ and 1^st^ person flying conditions. It is thought that the frontoparietal attention network can modulate brain activity in modality specific perceptual areas [55] of the brain. It is possible that the attention necessary for flying in the 3^rd^ person is greater than that of the 1^st^ and that this is what is reflected in the differential activity between the conditions. Both the parietal and premotor regions are thought to be involved with attention related processing [56], [57]. However, given that the flying task was difficult and required attention in both 1^st^ and 3^rd^ person conditions it is unlikely that the differential results found in our study (Figures 5A and 6B, Table 1 and 4) are just a result of general differential attentional modulation. Rather, we maintain that the differential brain activity is a result of neural processes involved with aspects of dynamic visuomotor transformation that may be specifically modulated by attention.

### Conclusion

This study set out to determine the neural processes underlying dynamic visuomotor transformation of the kind used during remote operation of vehicles. It was maintained that the ability for humans to be able to carry out remote control has its foundations in imitation learning. The primary finding implicating the ‘Mirror Neuron’ system related brain regions (IPL and PMv) suggests that this system is not only involved in processing goal directed action but also in visuomotor transformation processes that are also necessary for imitation. These finding extend the implication of the ‘Mirror Neuron’ system beyond that of body parts to include observation and manipulation of the action of objects (in this case an airplane). Just as Gibson [58] realized the importance of dynamic optic flow fields for ecological perception in reference to aviation research on pilots during approach to landing we believe that this field we call ‘Aviation Cerebral Experimental Sciences (ACES)’ will help to better understand the neural processes underlying complex perception, motor control, and cognitive functions. One of the primary strengths of this experiment was that the tasks involved complex perceptual motor control in a robust simulated environment in which clear hypothesis driven differential brain activity in relevant brain regions could be discerned. This is in contrast to most experiments that utilize tasks and stimuli that are far removed from real-world experience that only occur in a laboratory setting. The ACES approach is well suited to provide information regarding neural processes that is directly applicable to real-world situations. This is important for the development of practical brain-machine interface applications.

## Supporting Information

Movie S1 **Example of a trial in the first person F1 perspective.**
(MP4)

Movie S2 **Example of a trial in the third person F3 perspective.**
(MP4)
